# Leveraging large language models to maintain a branded food product database

**DOI:** 10.1038/s41538-026-00909-1

**Published:** 2026-06-16

**Authors:** Pascal Hauff, Carolin Krems, Jan Kohl

**Affiliations:** Department of Nutritional Behaviour, Max Rubner-Institut, Federal Research Institute of Nutrition and Food, Karlsruhe, Germany

**Keywords:** Computational biology and bioinformatics, Mathematics and computing

## Abstract

Branded food data are essential for assessing contemporary dietary behavior and the global food environment. However, processing such data is challenging due to its vast, rapidly changing nature, variable quality, and numerous sources. To address these limitations, we developed a fully automated, large language model (LLM)-powered pipeline for collecting, standardizing, and enriching branded food data, enabling ingredient-level analyses, and facilitating estimation of ingredient quantities and undeclared nutrient content. Evaluation of LLM performance demonstrated that a fine-tuned model outperformed the human experts in parsing and mapping product data. Non-fine-tuned LLMs showed insufficient performance, whereas even modest amounts of fine-tuning data substantially improved results. Overall, LLMs provide a scalable approach for processing branded food data and supporting more consistent and standardized data curation, with performance exceeding that of an individual human expert. These results highlight the potential of LLMs to transform the management and analysis of complex, large-scale food databases.

## Introduction

Accurate and comprehensive food composition data are essential for nutritional science, particularly for assessing nutrient intake and informing public health policies^1^. Generic food databases with standardized nutrient profiles for commonly consumed foods remain fundamental resources in nutrition research^1^, but their limited coverage of branded food products restricts relevance in dietary assessments.

Branded food databases can improve the realism and timeliness of dietary assessment by capturing specific food products rather than relying solely on generic food types with averaged composition data^2^. For example, branded food data facilitates the identification of specific ingredients such as added sugars and food additives, as well as details regarding preservation methods and packaging materials^2–4^.

The development of branded food databases has gained momentum across many countries in recent years^2,5–9^. However, the rapid turnover of the food market makes it impractical and costly to rely solely on chemical analyses for compositional data^7,10,11^. As an alternative, the composition of branded food products can be estimated based on mandatory food labeling, which provides ingredient statements and nutrient declarations^12^.

Despite its potential, using product labeling data poses several challenges in terms of data availability, quantity, and quality. Achieving adequate coverage often requires a combination of data acquisition methods, including automated approaches like web scraping and API access^2,7,13^, as well as manual approaches such as extracting information from manufacturer websites or photographing product packaging in stores or post-purchase^9,14^. Crowdsourced data collection, as used in countries like Slovenia and Australia^13,15^ and by international platforms such as Open Food Facts (https://world.openfoodfacts.org/) and Yuka (https://yuka.io/de/), provides an inexpensive alternative. However, such crowdsourcing shifts control over data quality and governance to non-professional contributors.

**Fig. 1:**
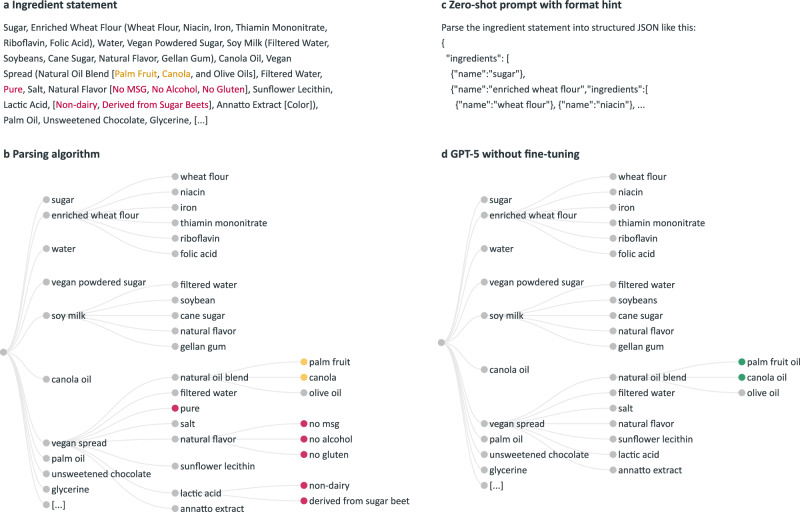
Example for limitations of algorithmic parsing. Parsing example of an ingredient statement (**a**) using an algorithm^18^ (**b**) and GPT-5 (**d**) on a zero-shot prompt with format hint (**c**). Errors in parsing, in the sense of incorrectly recognized ingredients, are shown in red. Contexts that have not been transferred are shown in yellow, while the correct transfer of context is shown in green.

Given the volume of branded food data and inconsistencies in terminology, spelling errors, and free-text entries^2^, purely manual curation is not feasible. Several efforts have explored partial automation of food label processing, primarily through rule-based parsing pipelines, curated thesauri and named-entity recognition (NER). For example, the US Food and Drug Administration described a parsing pipeline that uses manually constructed “remove/convert/preserve” registries to standardize separators, avoid incorrect splits, clean noise, and preserve nested structures^16^. This type of delimiter- and exception-driven parsing requires substantial manual resources to build and maintain registries and to correct manufacturer label errors, while the resulting parsed terms can still contain spelling variants and synonyms that require an additional synonym database. Similarly, the IngID framework combines extensive parsing with a thesaurus that maps equivalent ingredient strings (e.g., synonyms and spelling errors) to preferred descriptors, with the authors themselves stating the parsing methodology is cumbersome, time-consuming and not sustainable^17^. An example from a recent study (Fig. 1a, b) demonstrates how missing semantic understanding can lead to parsing errors^18^. The more advanced machine-learning approaches have largely framed ingredient statement parsing as named-entity recognition over ingredient expressions. Despite promising performance, they remain constrained by variation in input syntax, requirements for large amounts of manually annotated data and can suffer from dataset artifacts such as spurious correlations between token position and predicted labels^19^. Because such models typically output token-level tags rather than hierarchical ingredient lists, additional domain-specific post-processing and substantial manual work would be required to reconstruct nested compound ingredients from ingredient statements. Furthermore, these automation approaches lack generative capabilities, preventing them from resolving syntactic defects, reconstructing missing structural elements, inferring displaced semantic context or producing free-text such as streamlined food descriptions.

Considering these limitations, it is not surprising that branded food databases focus on basic quality checks (e.g., completeness and plausibility of nutrient values) and, when information is unclear, may exclude products, or contact manufacturers^2,6,9^. More sophisticated data processing remains limited, restricting the ability to derive deeper insights or ensure data comparability across sources.

Large language models (LLMs) offer a complementary approach that is better matched to the free-text and hierarchical nature of ingredient statements. Rather than relying on brittle delimiter logic or token-level tagging, an LLM can be prompted (and fine-tuned) to directly translate an ingredient statement into a schema-constrained, hierarchical JSON structure as demonstrated in Fig. 1c, d. For mapping, combining LLMs with retrieval-augmented generation (RAG) allows standardization to be framed as a constrained decision: for each parsed ingredient, a small set of semantically similar candidates from a controlled vocabulary is retrieved, and the model selects the best match (or, if necessary, proposes a new term), improving consistency while keeping the process auditable and updatable. In addition, LLMs can also output free-text, such as a meaningful product name and functions based on multiple GDSN attributes (e.g., functional descriptions, ingredient information, and marketing-related fields). This flexibility allows for agile pipeline development and adaptation to a wide range of use cases without dependency on large amounts of domain-specific training data.

This study presents a fully automated, LLM-powered pipeline for collecting, standardizing, and enriching branded food data and provides a systematic evaluation of its performance. We characterize the ability of an LLM to transform unstructured product labeling text into a consistent, structured representation suitable for large-scale database maintenance, and assess how the accuracy of an LLM compares to that of an individual domain expert when benchmarked against a consensus-based gold standard. In addition, we explore the data requirements associated with this performance level by examining how accuracy scales with the amount of fine-tuning data. Together, these analyses provide an empirical characterization of the performance and robustness of LLM-based automation for ingredient-level food data processing, informing its potential use in large-scale food environment analyses and dietary assessment.

## Results

### Development of an LLM-powered pipeline

**Fig. 2:**
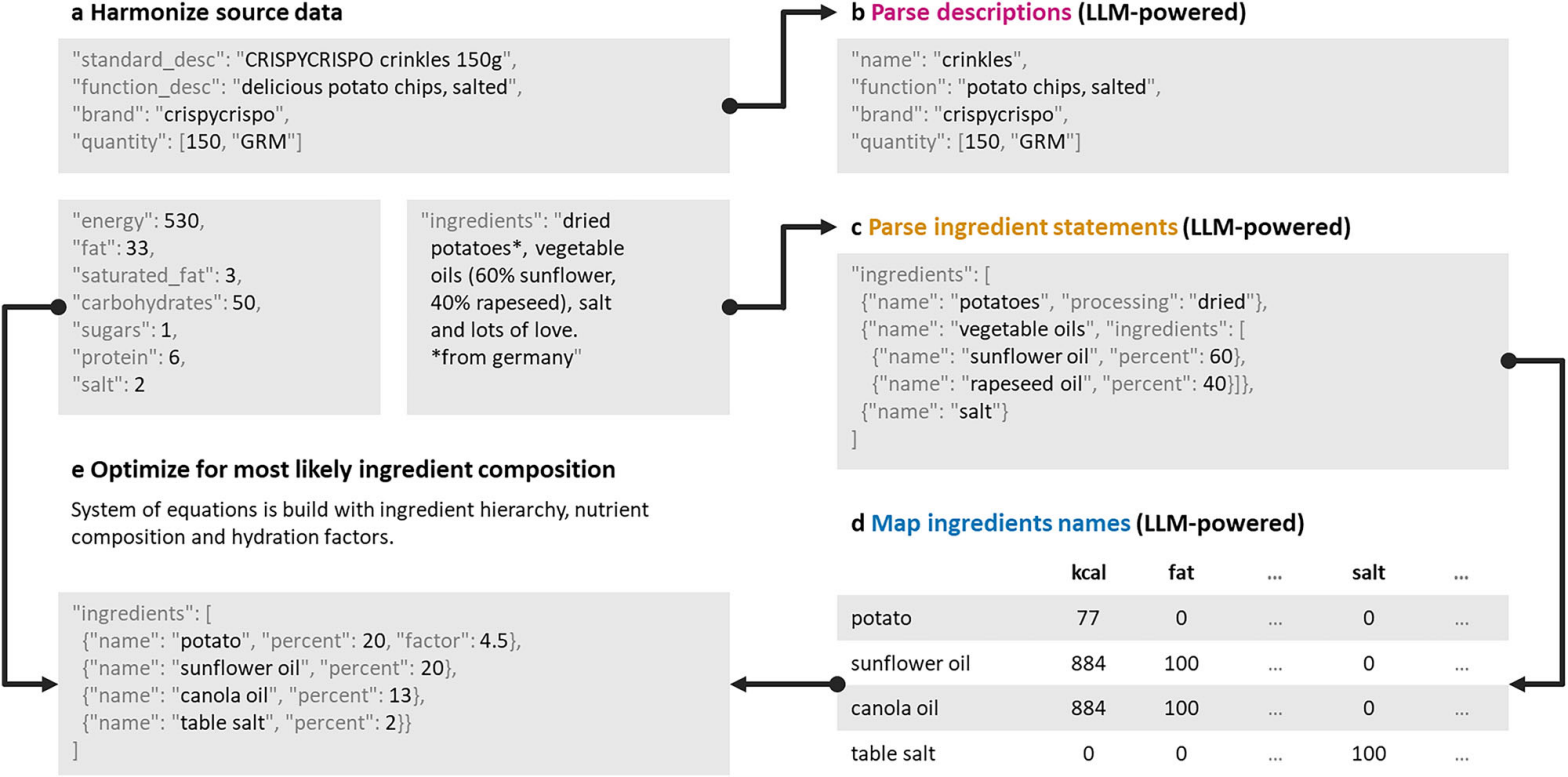
Overview of the LLM-powered pipeline. Flow chart showing sequential processing steps with simplified examples. The harmonization step (**a**) delivers data in a format that all subsequent steps operate on. The two parsing tasks (**b**, **c**) and the mapping task (**d**) involve the use of large language models (LLMs). The optimization step (**e**) uses the processed data to estimate compositional information using hydration factors to account for processing yield.

**Fig. 3:**
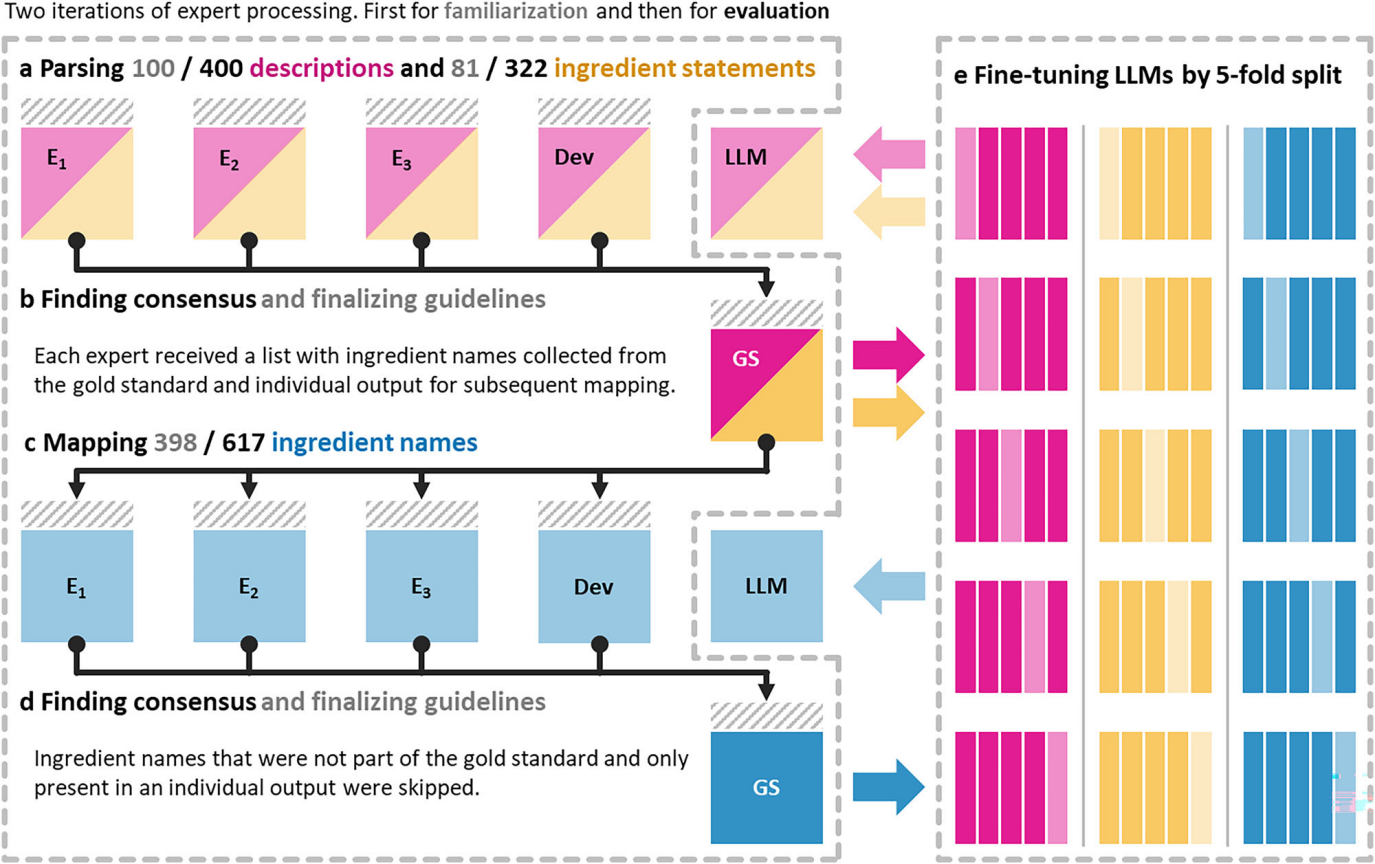
Overview of gold standard dataset creation. Three experts (E_1–3_) manually parsed and mapped 500 products, whereas the pipeline developer (Dev) manually reviewed output from now-outdated LLMs. The familiarization round (**a**–**d**) with 100 products was used to refine the guidelines. The evaluation round (**a**–**d**) with the remaining 400 products produced the gold standard (GS) for evaluation of the outputs. For each task, five LLMs were fine-tuned using a fivefold split of the gold standard (**e**). The transparent fifths indicate the data excluded from fine-tuning and only used for evaluation.

**Fig. 4:**
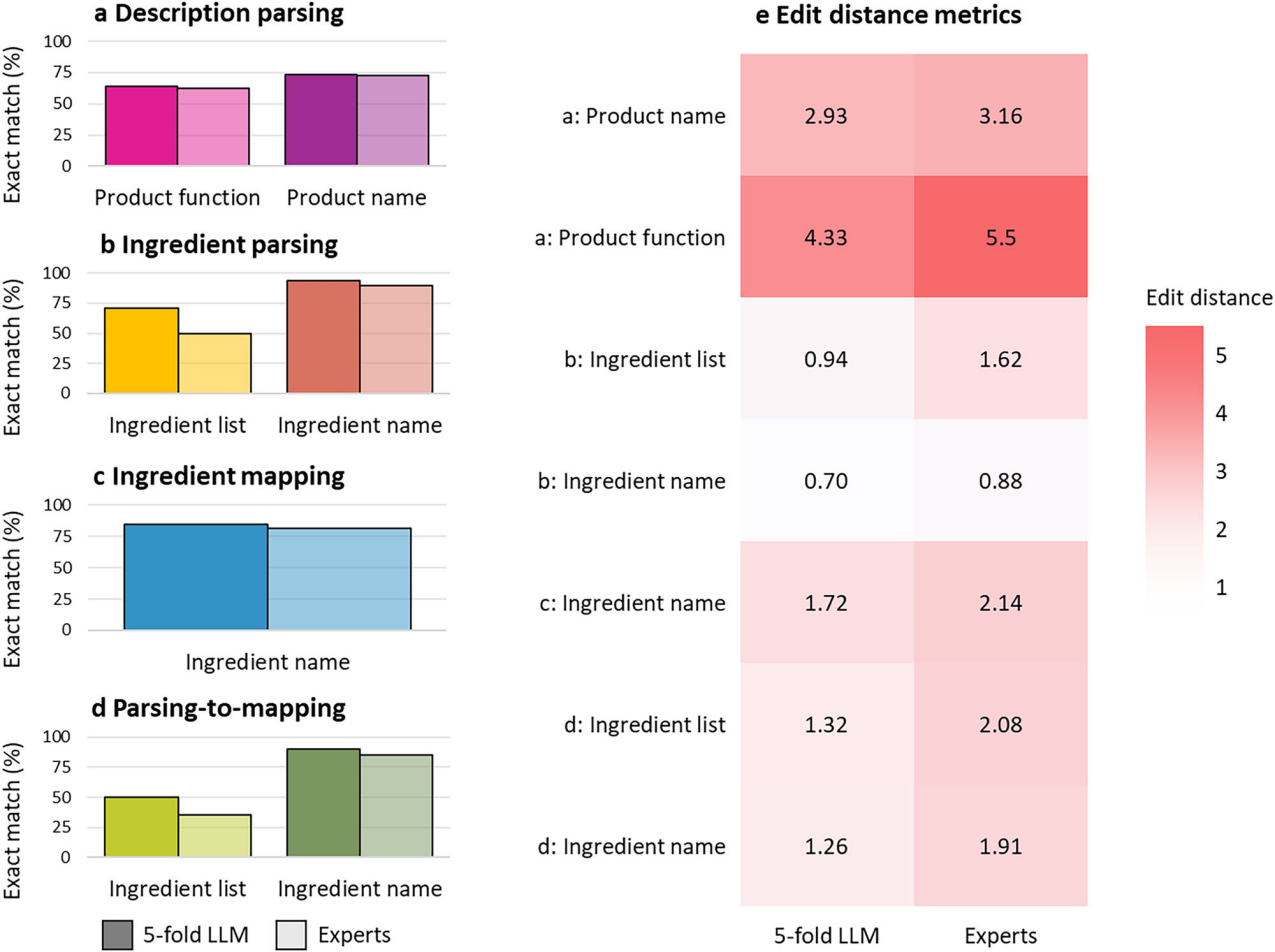
Performance in branded food data processing of LLM and experts. Performance relative to the gold standard of a fivefold LLM compared with experts (*n* = 3, arithmetic mean) in the sub-areas: **a** parsing of the product description, **b** parsing of the ingredient statement, **c** mapping of ingredients to the reference database, and **d** parsing-to-mapping of ingredients. **e** Heat map showing edit distance per product: DeepDiff for ingredient lists, Levenshtein for all other sub-areas. Error bars and brackets indicate 95% confidence intervals.

**Fig. 5:**
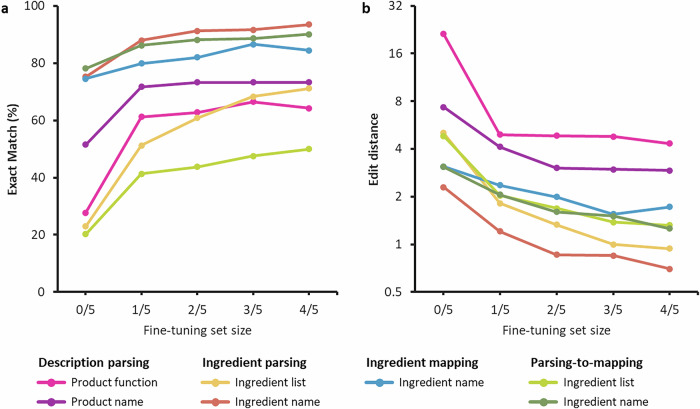
Impact of fine-tuning set size on LLM performance in parsing and mapping. Performance relative to the gold standard of LLMs (GPT-4.1 mini) with different amounts of fine-tuning data (0/5 = no fine-tuning, 4/5 = fivefold LLM). Shown are the exact match rate (**a**) and edit distance (**b**) across all sub-areas. The edit distance metric used was DeepDiff for ingredient lists, Levenshtein for all other sub-areas. Panel (**b**) uses a logarithmic scale to improve visibility of changes at lower error levels.

The data processing pipeline was developed with a focus on basic functionality to facilitate early testing. The data acquisition was limited to branded food data from the Global Data Synchronization Network (GDSN), which is a platform where numerous data providers along the supply chain exchange product information. This approach eliminated the need to merge data from multiple acquisition methods, while still providing very heterogeneous data quality to evaluate the robustness of the pipeline. The processing comprised four sequential steps: harmonization, parsing, mapping, and optimization (Fig. 2). The LLM-powered tasks are color-coded for Figs. 2–5. Additional acquisition methods were prototyped but are beyond the scope of this evaluation.

In the harmonization step (Fig. 2a), the source data is converted into a standardized JSON format for uniform processing. The GDSN exchanges data in XML format (https://gdsn.gs1-germany.de/attributes). We mapped the source XML fields to our JSON schema. After this one-time alignment, all subsequent processing operates on the standardized JSON, regardless of the data source.

In the parsing step (Fig. 2b, c), structured information is extracted from product descriptions and ingredient statements using an LLM. The description parsing (Fig. 2b) was performed with brand name and product quantity as context to extract a concise, standardized name and function of the product. The function serves as a fallback ingredient, creating a single-item ingredient list containing only the function itself when no ingredient statement is provided. It is required as the name does not necessarily indicate which food is contained (e.g., “Pig on a board with cutlery” as a name for a marzipan preparation). The ingredient parsing (Fig. 2c) was applied to each product’s ingredient statement to generate a structured ingredient list. Each ingredient object in the list includes a name and, when available, optional attributes:

- Percentage: The declared percentage of the ingredient in the product.
- Processing: The ingredient’s processing state.
- Ingredients: Any nested ingredients listed within a compound ingredient.

In the mapping step (Fig. 2d), the textual entries from the parsing output were linked to controlled vocabularies in reference databases using an LLM. The evaluation in this study focuses solely on ingredient mapping, where each parsed ingredient that has no nested ingredients (bottom-level), is mapped to the reference database to retrieve nutrient information.

In the optimization step (Fig. 2e), the complete composition of a product is inferred by adjusting unknown ingredient proportions to satisfy declared percentages and compositional constraints using a sequential least squares programming (SLSQP) solver, following the methodology of Bohn et al. (2022)^12^. The estimated nutrient composition is as close as possible to the declared nutrients from the product label and has to be within regulatory tolerances specified by the Regulation (EU) No 1169/2011 of the European Parliament.

### Evaluation of the LLM performance

To assess the performance of the LLM-powered pipeline, we compared its output for the two parsing tasks (Fig. 2b, c) and the mapping task (Fig. 2d) against a human-curated gold standard. We created the gold standard dataset through a multi-step consensus process involving a team of three experts (nutrition scientists) and the pipeline developer (Fig. 3), starting with a familiarization run on 100 sample products with preliminary guidelines.

The experts independently parsed the same 100 products manually, and the pipeline developer parsed the sample products using a combined approach of LLM outputs from now-outdated models fine-tuned on data curated in the development process (Fig. 3a). This combined approach represents iterative data curation under development conditions and was used to ensure the quality of the gold standard. All outputs were compared, discrepancies identified, and guidelines refined through consensus discussions (Fig. 3b).

Subsequently, a mapping test run was conducted on the ingredient names from the parsed ingredient lists (Fig. 3c). Each expert mapped the ingredient names to the reference database manually, whereas the pipeline developer employed the LLM-assisted approach. Again, consensus discussions further refined mapping guidelines based on identified discrepancies (Fig. 3d).

With the finalized guidelines, the team processed the remaining 400 products (Fig. 3a–d). Ambiguities and discrepancies were resolved collectively to produce a consistent, high-quality gold standard dataset, used as the reference for evaluation. On average, it took each expert 27 h and 16 min to process 400 food products. Finalizing the guidelines and reaching consensus on 400 processed products required another 11 h and 20 min of meetings with the whole team present.

The evaluation involved systematically comparing outputs against the curated gold standard. The LLM pipeline was represented by five GPT-4.1 mini models per task (Fig. 3e) fine-tuned in a fivefold cross-validation approach (“fivefold LLM”). This means that each model was fine-tuned on 4/5 of the gold standard dataset to then process the remaining 1/5 of the data, which it had never seen. Each output was compared to the gold standard, and the arithmetic mean for the experts was calculated to compare the performance of the fivefold LLM with the performance of the average expert.

The primary performance metrics for all tasks were the exact match rate and an adequate edit distance for counting the edits required to align an output with the gold standard. As secondary performance metrics for detailed comparison, we calculated F1 scores and embedding-based vector distance (100 - cosine similarity * 100). We estimated 95% confidence intervals using the bias-corrected and accelerated (BCa) bootstrap method. The fivefold LLM was closer to the gold standard than the experts based on each metric (Fig. 4 and Table 1). The performance across individual experts was comparable (Supplementary Tables 1–4).

**Table 1:** Performance in ingredient processing sub-areas of fivefold LLM and experts

Metric	Subarea	Fivefold LLM	Experts
**Description parsing**			
Vector distance	Product names	2.7 [2.2, 3.2]	3.4 [2.9, 4.0]
Vector distance	Product functions	3.0 [2.5, 3.6]	3.6 [3.1, 4.1]
**Ingredient parsing**			
Vector distance	Ingredient names	1.6 [1.0, 2.7]	2.2 [1.5, 3.1]
F1 score	Declared percentages	98.0 [95.4, 99.2]	97.7 [96.1, 98.5]
Precision		96.6 [91.5, 98.8]	98.1 [94.7, 99.3]
Recall		99.5 [98.2, 100.0]	97.3 [96.0, 98.3]
TPA/TPE/FP/FN		398/1/13/1	1165/1/22/31
F1 score	Processing states	89.3 [84.8, 92.9]	87.8 [83.8, 91.0]
Precision		91.6 [86.3, 95.3]	90.7 [85.8, 94.0]
Recall		87.1 [81.2, 91.8]	85.1 [80.3, 89.1]
TPA/TPE/FP/FN		142/4/9/17	430/10/34/65
F1 score	Bottom-level ingredients	93.0 [89.8, 95.1]	88.2 [85.3, 90.1]
Precision		93.0 [89.7, 95.1]	88.2 [85.4, 90.1]
Recall		93.0 [89.8, 95.1]	88.1 [85.3, 90.1]
TPA/TPE/FP/FN		3064/215/14/14	8707/1122/44/50
F1 score	Nesting	94.1 [90.8, 96.2]	92.6 [89.8, 94.7]
Precision		93.8 [89.0, 96.7]	90.9 [86.0, 94.0]
Recall		94.4 [90.9, 96.8]	94.4 [91.8, 96.2]
TPA/FP/FN		287/19/17	857/86/51
**Mapping**			
Vector distance	Ingredient name	2.2 [1.8, 2.8]	2.7 [2.3, 3.1]
F1 score	Ingredient name (retrieval only)	90.2 [87.7, 92.1]	86.6 [84.4, 88.4]
Precision		89.3 [86.5, 91.7]	84.6 [82.1, 87.0]
Recall		91.1 [88.4, 93.3]	88.5 [86.3, 90.4]
TPA/TPE/FP/FN		501/27/33/22	1461/130/135/59
**Parsing-to-mapping**			
Vector distance	Ingredient names	2.4 [1.7, 3.5]	3.3 [2.6, 4.3]
F1 score	Bottom-level ingredients	89.7 [86.6, 91.7]	84.3 [81.6, 86.2]
Precision		89.7 [86.5, 91.7]	84.4 [81.7, 86.2]
Recall		89.7 [86.7, 91.7]	84.3 [81.6, 86.2]
TPA/TPE/FP/FN		2954/325/14/14	8330/1499/44/50

Experts (*n* = 3). Values are reported as arithmetic means; confusion matrix entries are reported as sums.

*TPA* true positive accurate, *TPE* true positive error, *FP* false positive, and *FN* false negative.

When parsing descriptions, the fivefold LLM performed only slightly better than the experts, except for a descriptively lower Levenshtein edit distance regarding product functions (Fig. 4a, e). When parsing ingredients, the fivefold LLM had a higher exact match rate than the experts (71.1% [66.1%, 75.8%] vs. 49.6% [45.3%, 53.8%]) and a lower DeepDiff edit distance (Fig. 4b, e). For ingredient names at the bottom level, the exact match rate (93.4% [90.3%, 95.4%] vs. 89.3% [86.5%, 91.1%]) and the Levenshtein edit distance were similar.

The ingredient mapping performance of the fivefold LLM and the experts was comparable in exact match rate (84.4% [81.4%, 87.0%] vs. 81.5% [78.8%, 84.0%]) and Levenshtein distance (Fig. 4c, e). When considering the parsing-to-mapping pipeline of ingredients, the cumulative effect of deviations resulted in a lower degree of conformity with the gold standard (Fig. 4d, e). The fivefold LLM achieved 50.0% [44.7%, 55.6%] exact matches, whereas the experts averaged 35.6% [31.7%, 39.9%] exact matches. For ingredient names at the bottom level, the conformity decreased less, with the fivefold LLM achieving 90.1% [87.1%, 92.0%] exact matches and the experts averaging 84.7% [82.1%, 86.6%] exact matches.

### Impact of fine-tuning and model size on LLM performance

In addition to the fivefold split with maximum fine-tuning data usage, we evaluated scenarios with less fine-tuning data (no fine-tuning and fine-tuning with one, two, or three fifths) and two additional GPT models—one larger (GPT-4.1), one smaller (GPT-4.1 nano) than GPT-4.1 mini—without fine-tuning. The performance of GPT-4.1 mini was the worst without fine-tuning and demonstrated an upward trend as the amount of fine-tuning data increased (Fig. 5 and Supplementary Tables 5–8). For ingredient parsing-to-mapping, a fifth of the available fine-tuning data led to more than a doubling of performance to an exact match rate of 41.3% and a DeepDiff distance of 2.03. The incorporation of additional fifths as fine-tuning data resulted in an enhancement of the exact match rate to 43.8, 47.5, and the previously documented 50.0%, while the distance decreased to 1.69, 1.38, and 1.32, respectively. GPT-4.1 outperformed GPT-4.1 mini across all sub-areas except for product functions, and GPT-4.1 nano was outperformed by GPT-4.1 mini across all sub-areas (Supplementary Tables 5–8).

## Discussion

Our objective was to assess whether automated processing of branded food data can replace the labor-intensive manual procedures currently required to support ingredient-level analyses. We therefore evaluated the extent to which an LLM-based pipeline can transform unstructured product labeling text into structured representations, and how its accuracy compares to that of an individual domain expert when benchmarked against a consensus-based gold standard, as well as how this performance scales with the amount of fine-tuning data.

We observed that individual experts deviate heavily from the gold standard (Fig. 3b, d). This suggests that the involvement of multiple experts, along with consensus discussions, is required to ensure high data quality. Based on the time spent on creating the gold standard dataset, processing 100,000 products simultaneously would take about 6750 h (about 3 years of full-time work) per expert. In consequence, purely manual processing is not feasible. The alternative we present is using the manual approach to create a gold standard dataset to subsequently fine-tune LLMs for fully automated branded food data processing.

The fivefold LLM already outperformed the experts by all metrics with relatively little fine-tuning data. We observed the most notable advantage for the central task of ingredient parsing, and varying fine-tuning set sizes indicate the most potential for further improvement with more fine-tuning data. Other sub-areas exhibit indications of overfitting, accompanied by a decline in performance. As far as we could observe with GPT-4.1, larger models cannot simply be used to circumvent the need for fine-tuning.

Despite outperforming the experts, the fivefold LLM still showed substantial deviations from the gold standard based on the metrics used in this study. This is a result of sensitive metrics as well as complex tasks, which resulted in an elevated error rate in both LLMs and experts. In many cases, deviations from the gold standard are only superficial in nature and can be considered semantically equivalent, which is expressed in low mean vector distances for description parsing and ingredient mapping. For example, the fivefold LLM added “dextrose” to the reference database, which already contained the synonymous “glucose”, whereas in the gold standard, it was always mapped to the existing “glucose”. Both ingredients refer to the same nutrient composition data, yet this superficial deviation affects 44 parsing-to-mapping ingredient lists and accounts for 10.8% of the average edit distance. Mapping directly to the German food composition database^20^ instead of a more comprehensive and expandable reference database would lead to fewer deviations. However, this would lead to the loss of ingredient information by grouping prematurely.

The greatest deviation by edit distance was evident in description parsing. This can only partly be attributed to the average name (20 letters) and function (42 letters) being longer than the average ingredient name (17 letters). It is also a consequence of ambiguity regarding which parts of available descriptions belong to the product name and which to the product function. For example, the two descriptions “TOWER Milk Break—Iced Coffee 500 ml” and “TOWER Milk Break—Iced Café Drink 500 ml” were provided for one product, making it ambiguous whether “Milk Break—Iced Coffee” or “Milk Break—Iced Café Drink” is the product name on the package. Such variations in product names do not prevent product identification (e.g., by participants in nutrition surveys), and variations in product functions can still lead to the same mapping. Therefore, deviations from the description parsing gold standard cannot be considered errors by default.

In contrast, hierarchy deviations in ingredient lists mostly represent objective violations of the parsing guidelines. They are regularly serious errors as fundamental mathematical constraints in the optimization change due to the altered order and nesting. These deviations occur particularly frequently in food additives, as the grouping in combination with the functional description is ambiguous in many ingredient lists. Since additives play a minor role in optimization due to their low contribution, it is more relevant to be able to assess whether specific food additives are present. Based on the observed deviations in this study, streamlining the guidelines regarding additives (e.g., by not prohibiting the nesting of single ingredients) would reduce hierarchy deviations.

The presented methodology and pipeline are subject to external and internal limitations. Current food labeling regulations exhibit significant gaps in ingredient transparency. Compound ingredients comprising less than 2% of a product are exempt from nested ingredient declaration requirements, creating opacity in compositional analysis. Then there are ingredients like meat that are often not specified further than the animal type, but have high compositional variance. Last, there are fermented foods (e.g., beer) that, even when declared at the ingredient level, should not be simplified to the sum of their ingredients (e.g., water, malt, and hop)^12^.

Concerning internal limitations, the approximate-nearest neighbor index (ANN-index) for the mapping task was built only with self-mappings from the ingredient reference database and E-number-mappings of the compact representation (e.g., E101) to the reference database representation (e.g., E101 Riboflavins (G): (i) Riboflavin and (ii) Riboflavin-5-phosphate). Outside the context of a controlled evaluation, matches from existing fine-tuning data would be used to enhance RAG functionality. Also concerning ingredient mapping, the reference database lacks granular classification of generic foods (e.g., classifying grapes as berries with pulp, skin, and seeds). This complicates data-driven decisions on when to extend the reference database and when to use a similar existing ingredient to map to (e.g., is grape pulp worth a new entry or should it be mapped to whole grapes).

Finally, it is important to emphasize that both our gold standard and the reported performance estimates are conditional on a specific task definition. Our evaluation targets German-language GDSN products, a fixed JSON output schema, and a particular controlled vocabulary and mapping policy. Modifying the output schema (e.g., adding or redefining attributes), changing guideline conventions, narrowing the product scope to a specific category, or switching to a different controlled vocabulary can shift the distribution of ambiguity and error patterns.

Given these limitations, the further development of generic and branded food databases should be more tightly integrated in the future. Missing ingredients in generic food databases hinder the accurate mapping of branded food ingredients to their corresponding counterparts in generic food databases^21^. Therefore, mapping to a more comprehensive ingredient reference database than the corresponding generic food databases is important in order to be able to identify corresponding gaps in a data-driven manner. Furthermore, the reference database should be designed to support adequate handling of undeclared composite (e.g., 1% ketchup), high variance (e.g., pig meat), and fermented ingredients (e.g., cheese [milk, lab]). This might allow for strict mapping to existing ingredients without the optional extension of the reference database, which could substantially increase mapping performance. All proposed improvements should be evaluated individually using an up-to-date gold standard dataset for data-driven decision making.

The current pipeline is already useful for accelerating branded food data curation, but it does not yet achieve the level of agreement with the human-curated gold standard that would justify fully autonomous deployment. Further development should therefore remain iterative and human-guided, with automation used primarily to accelerate the generation of additional fine-tuning data and the curation of the reference database, rather than relying on routine per-item review, which would not be scalable for large branded food datasets. Based on the error patterns identified in this study, future work should further refine the task definition, auxiliary resources, and modeling strategy, including experimenting with prior classification (e.g., FoodEx2^22^) of products into narrower domains (e.g., wine or filled pasta) to support more specialized models. Once these methodological improvements have been implemented, broader benchmarking across LLMs should be conducted using an updated gold standard. If feasible, such comparisons should also include a model developed specifically for this task rather than relying solely on fine-tuning of a general-purpose model.

In conclusion, the fine-tuned LLM-powered pipeline developed in this study demonstrates substantial potential to enhance branded food data processing by achieving greater accuracy and scalability than individual experts. LLM-powered pipelines can therefore play a central role in improving both the accuracy and efficiency of large-scale food data analysis. Future efforts should focus on expanding and refining auxiliary data to further strengthen the pipeline’s performance and integrating additional data sources.

## Methods

### Data source and sample

We randomly sampled a total of 500 consumer unit products from the food/beverage segment of the GDSN with Germany as the target market. Most attributes are already provided in well-structured, code-listed fields. However, there are free-text attributes critical for data enrichment and therefore require special attention during processing.

### Data processing

All code was written in Python (version 3.11). The LLMs were accessed via the OpenAI API (version 1.97.0) using the “/v1/chat/completions” endpoint with the model temperature set to zero. We constructed the prompts with the content of the guidelines finalized in the familiarization phase by the experts. The attributes (GDSN Code in brackets) relevant to LLM processing were tradeItemDescription (M259), descriptionShort (M260), functionalName (M253), regulatedProductName (M261), brandName (M254), netContent (M281) and ingredientStatement (M047).

For parsing, we used a system prompt containing the task and the output JSON schema alongside a user prompt containing the free-text content.

“Return a consumer-friendly product name and a food database-friendly function name. Exclude brand name and content quantity from the provided description. Ensure the output is a JSON object directly following the given schema without additional wrappers.”

For the more complex task of ingredient parsing, the prompt included a set of rules. These were imposed to make the output as deterministic as possible and avoid information overlap with other code-listed attributes, such as allergens.

*“Convert the German ingredients list into a JSON object following the schema below. Ensure strict adherence to formatting rules without additional wrappers*.

Rules:

- *Follow proper capitalization according to German grammar*.
- *Always use the singular form, even if uncommon, except for the German “Kräuter”*.
- *Do not use brackets in names (e.g., “starch (corn)”* → *“cornstarch”)*.
- *For E-numbers with names, do not use a space between the “E” and the number and put the number before the name (e.g., “E330 citric acid”)*.
- *Use commas only in chemical names or numeric values (e.g., “butter, fat reduced”* → *“butter fat reduced”)*.
- *Exclude allergen warnings (e.g., “contains milk”)*.
- Exclude non-nutritional remarks (e.g., “*fair trade”).
- *Exclude function descriptions for bottom-level ingredients (e.g., “acidifier citric acid”* → *“citric acid”)*.
- *Exclude shape descriptors that do not impact nutritional content (e.g., “tomato slices”* → *“tomato”)*.
- *Do not nest single ingredients*.
- *List cooking and drying methods separately, except for:*
  ○ *grain drying*
  ○ *dairy heating*
  ○ *proper names (e.g., “Johannisbrotkernmehl”)*
- *Add context to nested ingredients when necessary for identification, except for processing attributes (e.g., “flour[wheat, rye]”* → *“flour[wheat flour, rye flour]”)*.
- *Default to “fat” when categorizing oils and fats if both terms apply (e.g., “palm fat”, “copra fat”, “cocos fat”, “shea fat”).”*

For mapping of ingredient names, the mapping LLM was prompted to select one of the 15 closest ingredients from the reference database using cosine similarity. The approximate-nearest-neighbor (ANN) index was constructed with the hnswlib (version 0.8.0) package. The required embeddings were created with the OpenAI model text-embedding-3-large (3072 dimensions) that offers high semantic precision at the cost of speed using the “/v1/embeddings” endpoint. The ingredient reference database itself contained a controlled selection of ingredients with nutrient information from the German Nutrient Database (version 3.02)^20^. The used prompt included the option to add a new entry to the reference database if retrieval was deemed suboptimal.

*“Match the provided German ingredient (string) with the most similar of the provided nearest neighbors. The context is the mapping of ingredients to foods from a nutrient database. Make sure to either respond with a string from the nearest neighbors or—if there is no good match—a new matching ingredient. Respond without any additional text.”*

If the LLM returned a neighbor, the ingredient was mapped accordingly. If it returned a novel term, that term was added to the ingredient reference database and immediately used for the mapping. Other attributes (e.g., units) were also normalized by mapping them to controlled vocabularies, but do not require LLM usage as code lists are available from the GDSN documentation.

### Evaluation

The exact match rate represents the percentage of outputs exactly matching the gold standard, with an exception for capitalization. The Levenshtein distance calculated with the levenshtein package (version 0.27.1) was used to compare text attributes and represents the minimum number of character edits required to transform the output to the gold standard. To provide a semantically-aware complement to Levenshtein distance, we additionally computed the vector distance using the SciPy package (version 1.17.0). The required text embeddings of the compared strings were created with the model text-embedding-3-large from OpenAI. The DeepDiff tree edit distance calculated with the deepdiff package (version 8.4.2) was used to compare entire ingredient lists and represents the number of attributes that must be changed to transform the output to the gold standard. With both edit distances, zero equals an exact match.

To get further insight into differences identified by the DeepDiff module and the ingredient mapping RAG system, F1 scores were calculated along with Precision and Recall. The F1 score is a harmonic mean of precision and recall, providing a balanced measure of accuracy. Precision is the proportion of correctly identified attributes among all attributes predicted by the model. Recall is the proportion of correctly identified attributes out of all attributes present in the gold standard.

Point estimates were reported together with 95% confidence intervals, which were computed with SciPy using non-parametric bias-corrected and accelerated (BCa) bootstrap resampling with 10,000 resamples at the product level. Given the heterogeneous complexity of ingredient lists, these intervals are intended to describe variability within the dataset and should not be interpreted as population-level uncertainty.

Due to the complex nature of the nested ingredient lists, the same difference can be interpreted differently. For example, if an output is missing an entire level of nested ingredients, it can be viewed as one instance of missing nesting or multiple ingredient deletions. Since the DeepDiff module determines the minimum number of edits, it would interpret it as the former. In consequence, different error patterns require different comparisons. Nesting errors were determined by comparing the standard nested ingredient lists. Name, percentage, and processing attributes were compared in an unordered flat representation of the ingredient list to avoid counting errors already falling under another error pattern. Lastly, the bottom-level ingredients were compared separately in an ordered flat representation of the ingredient list, as these are the ones to be mapped to the reference database and therefore of especially high relevance.

All F1 score calculations require counting true positives (TP), false positives (FP), and false negatives (FN). To capture the complex nature of the tasks, true positive accurate (TPA) and true positive error (TPE) were differentiated. TPE were treated as both FP and FN. Below are the definitions by attribute:

- Declared percentage and processing state attributes
  ○ **TPA:** No edit needed
  ○ **TPE:** Value change required
  ○ **FP:** Attribute needs to be deleted
  ○ **FN:** Attribute needs to be inserted
- Bottom-level ingredients
  ○ **TPA:** No edit needed
  ○ **TPE:** Name change required
  ○ **FP:** Ingredient object needs to be deleted
  ○ **FN:** Ingredient object needs to be inserted
- Nesting
  ○ **TPA:** Ingredient list is present in the output and the gold standard
  ○ **FP:** Ingredient list needs to be deleted
  ○ **FN:** Ingredient list needs to be added
- Ingredient name (retrieval only)
  ○ **TPA:** Match to existing entry in output and gold standard; correct match
  ○ **TPE:** Match to existing entry in output and gold standard; wrong match
  ○ **FP:** Match to existing entry in output, but new insertion in gold standard
  ○ **FN:** New insertion in output, but match to existing entry in gold standard

## Supplementary information

Supplementary table

## Data Availability

All processing outputs generated during this study are available for download via Zenodo at 10.5281/zenodo.18605234 (ref. ^23^).

## Code availability

Code to replicate the data analysis is available for download via Zenodo at 10.5281/zenodo.18605234 (ref. ^23^).
