# Supplementary material for: Leveraging large language models to maintain a branded food product database
Source: NPJ Sci Food. 2026 Jun 16;10:191. doi: 10.1038/s41538-026-00909-1 (PMC13273058; doi:10.1038/s41538-026-00909-1)
Supplement: Supplementary file 1 — Supplementary table [file 41538_2026_909_MOESM1_ESM.pdf]

Supplementary information

Supplementary Table 1: Individual expert results for description parsing

Metric	Sub-area	Expert 1	Expert 2	Expert 3
Exact match (%)	Product names	74.5 [70.0, 78.5]	69.8 [65.0, 74.0]	72.2 [67.5, 76.5]
Levenshtein		2.9 [2.3, 3.6]	3.4 [2.8, 4.1]	3.2 [2.5, 4.0]
Vector distance		3.1 [2.5, 3.8]	4.0 [3.3, 4.9]	3.0 [2.4, 3.8]
Exact match (%)	Product functions	64.5 [59.8, 69.0]	61.5 [56.8, 66.2]	60.2 [55.5, 65.0]
Levenshtein		4.8 [3.9, 6.1]	5.6 [4.5, 7.4]	6.0 [4.8, 8.1]
Vector distance		2.9 [2.4, 3.5]	3.5 [2.9, 4.2]	4.4 [3.7, 5.1]

Supplementary Table 2: Individual expert results for ingredient parsing

Metric	Sub-area	Expert 1	Expert 2	Expert 3
Exact match (%)	Ingredient list	42.5 [37.3, 48.1]	55.3 [49.7, 60.6]	50.9 [45.3, 56.2]
DeepDiff		1.9 [1.6, 2.3]	1.6 [1.3, 2.0]	1.4 [1.2, 1.7]
Exact match (%)	Ingredient names	88.6 [85.6, 90.6]	90.3 [86.3, 92.8]	88.9 [84.8, 91.7]
Levenshtein		0.8 [0.6, 1.1]	0.8 [0.5, 1.2]	1.1 [0.8, 1.5]
Vector distance		1.6 [1.0, 2.7]	2.1 [1.3, 3.5]	2.7 [1.8, 4.1]
F1 score	Declared percentages	98.1 [96.8, 98.9]	96.6 [93.7, 98.2]	98.4 [95.7, 99.3]
Precision		99.2 [98.0, 99.8]	96.5 [90.9, 99.0]	98.5 [92.5, 100.0]
Recall		97.0 [94.7, 98.4]	96.7 [94.4, 98.3]	98.2 [96.5, 99.3]
TPA/TPE/FP/FN		386/0/3/12	386/0/14/13	393/1/5/6
F1 score	Processing states	90.1 [86.2, 93.1]	84.6 [77.3, 89.4]	89.0 [84.4, 92.4]
Precision		97.9 [94.2, 99.4]	85.4 [74.0, 91.5]	89.8 [83.9, 94.0]
Recall		83.4 [77.3, 88.5]	83.8 [77.7, 89.1]	88.2 [82.6, 92.3]
TPA/TPE/FP/FN		141/0/3/28	140/7/17/20	149/3/14/17
F1 score	Bottom-level ingredients	87.4 [84.3, 89.4]	89.6 [85.5, 92.1]	87.5 [83.4, 90.4]
Precision		87.5 [84.4, 89.5]	89.5 [85.5, 92.1]	87.6 [83.4, 90.4]
Recall		87.3 [84.2, 89.4]	89.6 [85.5, 92.2]	87.5 [83.3, 90.4]
TPA/TPE/FP/FN		2876/405/7/12	2951/323/22/19	2880/394/15/19
F1 score	Nesting	93.3 [90.1, 95.5]	91.6 [87.7, 94.3]	92.9 [89.7, 95.2]
Precision		92.6 [87.9, 95.5]	89.5 [83.5, 93.5]	90.6 [85.5, 94.2]
Recall		94.1 [90.1, 96.7]	93.7 [90.3, 96.1]	95.4 [92.4, 97.4]
TPA/FP/FN		286/23/18	282/33/19	289/30/14

True positive accurate (TPA), true positive error (TPE), false positive (FP), and false negative (FN)

Supplementary Table 3: Individual expert results for ingredient mapping

Metric	Sub-area	Expert 1	Expert 2	Expert 3
Exact match (%)	Ingredient name	80.4 [77.1, 83.5]	84.1 [81.0, 86.9]	79.9 [76.5, 83.0]
Levenshtein		2.1 [1.7, 3.2]	1.7 [1.3, 3.0]	2.6 [2.1, 3.8]
Vector distance		2.8 [2.3, 3.4]	2.2 [1.8, 2.7]	3.1 [2.5, 3.7]
F1 score	Ingredient name (retrieval only)	86.3 [83.6, 88.8]	88.8 [86.3, 90.9]	84.5 [81.6, 87.1]
Precision		87.1 [84.1, 89.7]	86.2 [83.1, 88.8]	81.0 [77.7, 84.0]
Recall		85.6 [82.5, 88.3]	91.6 [89.0, 93.7]	88.4 [85.5, 90.9]
TPA/TPE/FP/FN		471/42/28/37	504/32/49/14	486/56/58/8

True positive accurate (TPA), true positive error (TPE), false positive (FP), and false negative (FN)

Supplementary Table 4: Individual expert results for parsing-to-mapping

Metric	Sub-area	Expert 1	Expert 2	Expert 3
Exact match (%)	Ingredient list	31.7 [26.7, 37.0]	45.0 [39.8, 50.6]	30.1 [25.5, 35.4]
DeepDiff		2.2 [1.9, 2.6]	1.8 [1.5, 2.2]	2.2 [1.9, 2.6]
Exact match (%)	Ingredient names	85.0 [82.1, 86.8]	88.4 [84.6, 90.7]	80.9 [77.2, 83.7]
Levenshtein		1.5 [1.3, 1.9]	1.6 [1.1, 2.3]	2.6 [2.2, 3.3]
Vector distance		2.7 [2.1, 3.8]	2.7 [1.8, 4.1]	4.5 [3.5, 5.9]
F1 score	Bottom-level ingredients	84.8 [81.8, 86.6]	87.8 [84.0, 90.2]	80.5 [76.7, 83.3]
Precision		84.8 [81.9, 86.7]	87.8 [84.0, 90.2]	80.5 [76.7, 83.3]
Recall		84.7 [81.7, 86.6]	87.9 [84.1, 90.3]	80.4 [76.6, 83.3]
TPA/TPE/FP/FN		2789/492/7/12	2893/381/22/19	2648/626/15/19

True positive accurate (TPA), true positive error (TPE), false positive (FP), and false negative (FN)

Supplementary Table 5: Description parsing performance of GPT-4.1 mini with varying training data sizes (0/5 = no fine-tuning, 4/5 = 5-fold LLM) vs. non-fine-tuned GPT-4.1 nano and GPT-4.1

Metric	Sub-area	GPT-4.1 mini					GPT-4.1 nano	GPT-4.1
		**0/5 No fine-tuning**	**1/5 fine-tuning set size**	**2/5 fine-tuning set size**	**3/5 fine-tuning set size**	**4/5 fine-tuning set size**	**No fine-tuning**	**No fine-tuning**
Exact match (%)	Product names	51.5 [46.8, 56.5]	71.8 [67.2, 76.0]	73.2 [68.8, 77.5]	73.2 [68.8, 77.5]	73.2 [68.8, 77.5]	15.5 [12.0, 19.2]	53.2 [48.2, 58.2]
Levenshtein		7.4 [6.4, 8.5]	4.1 [3.3, 5.3]	3.0 [2.4, 3.8]	3.0 [2.4, 3.7]	2.9 [2.3, 3.6]	13.5 [12.6, 14.4]	7.0 [6.0, 8.1]
Vector distance		6.0 [5.2, 6.9]	3.6 [3.0, 4.4]	3.0 [2.4, 3.6]	2.9 [2.4, 3.5]	2.7 [2.2, 3.2]	14.0 [13.0, 15.0]	6.2 [5.3, 7.1]
Exact match (%)	Product functions	27.8 [23.5, 32.2]	61.3 [56.5, 66.0]	62.7 [58.0, 67.2]	66.5 [61.8, 71.0]	64.2 [59.5, 68.8]	1.5 [0.5, 3.2]	7.8 [5.5, 10.8]
Levenshtein		21.2 [18.9, 24.0]	4.9 [4.1, 6.2]	4.8 [4.0, 5.9]	4.8 [3.9, 5.9]	4.3 [3.5, 5.4]	35.5 [33.0, 38.6]	26.8 [24.4, 29.9]
Vector distance		13.1 [12.0, 14.2]	3.9 [3.3, 4.7]	3.6 [3.0, 4.3]	3.2 [2.7, 3.9]	3.0 [2.5, 3.6]	25.9 [25.0, 26.9]	14.7 [13.9, 15.6]

Supplementary Table 6: Ingredient parsing performance of GPT-4.1 mini with varying training data sizes (0/5 = no fine-tuning, 4/5 = 5-fold LLM) vs. non-fine-tuned GPT-4.1 nano and GPT-4.1

Metric	Sub-area	GPT-4.1 mini					GPT-4.1 nano	GPT-4.1
		**0/5 No fine-tuning**	**1/5 fine-tuning set size**	**2/5 fine-tuning set size**	**3/5 fine-tuning set size**	**4/5 fine-tuning set size**	**No fine-tuning**	**No fine-tuning**
Exact match (%)	Ingredient list	23.0 [18.6, 27.6]	51.2 [45.8, 56.8]	60.9 [55.6, 66.1]	68.3 [63.0, 73.3]	71.1 [66.1, 75.8]	17.4 [13.4, 21.7]	28.3 [23.6, 33.2]
DeepDiff		5.0 [4.4, 5.8]	1.8 [1.5, 2.2]	1.3 [1.1, 1.6]	1.0 [0.8, 1.4]	0.9 [0.7, 1.3]	6.7 [5.9, 7.5]	4.2 [3.7, 4.9]
Exact match (%)	Ingredient names	75.2 [71.3, 78.5]	87.9 [83.7, 90.9]	91.3 [87.3, 93.7]	91.6 [87.8, 94.2]	93.4 [90.3, 95.4]	64.3 [59.4, 68.6]	78.6 [75.4, 81.3]
Levenshtein		2.3 [1.9, 2.7]	1.2 [0.9, 1.7]	0.9 [0.6, 1.3]	0.9 [0.6, 1.3]	0.7 [0.5, 1.0]	3.1 [2.6, 3.6]	2.1 [1.8, 2.5]
Vector distance		4.7 [3.6, 6.0]	2.6 [1.7, 4.0]	2.0 [1.2, 3.4]	2.2 [1.3, 3.5]	1.6 [1.0, 2.7]	6.0 [4.7, 7.8]	3.7 [2.9, 4.9]
F1 score	Declared percentages	98.2 [96.2, 99.1]	97.0 [94.4, 98.4]	96.6 [93.7, 98.2]	97.5 [94.9, 98.9]	98.0 [95.4, 99.2]	97.2 [94.8, 98.6]	97.9 [96.1, 98.9]
Precision		97.3 [93.5, 98.9]	94.8 [90.0, 97.2]	94.1 [88.9, 97.0]	96.4 [91.5, 98.5]	96.6 [91.5, 98.8]	96.8 [93.0, 98.8]	97.6 [93.5, 99.0]
Recall		99.0 [97.7, 99.7]	99.3 [98.0, 99.8]	99.2 [97.9, 99.8]	98.8 [95.4, 99.7]	99.5 [98.2, 100.0]	97.6 [94.3, 99.2]	98.3 [96.8, 99.3]
TPA/TPE/FP/FN		404/1/10/3	404/2/20/1	396/2/23/1	396/1/14/4	398/1/13/1	240/0/8/6	404/1/9/6
F1 score	Processing states	44.9 [37.3, 53.8]	79.2 [72.8, 84.4]	81.6 [75.4, 86.7]	90.9 [86.8, 93.9]	89.3 [84.8, 92.9]	48.6 [34.9, 68.2]	48.3 [40.1, 56.4]
Precision		37.9 [30.3, 47.0]	85.1 [77.6, 90.5]	87.5 [79.9, 92.5]	96.6 [92.4, 98.8]	91.6 [86.3, 95.3]	47.7 [33.3, 68.6]	44.5 [35.1, 54.1]
Recall		55.1 [46.2, 64.6]	74.1 [66.5, 80.7]	76.4 [68.6, 83.2]	85.7 [79.6, 90.4]	87.1 [81.2, 91.8]	49.5 [34.0, 69.7]	52.8 [43.6, 61.8]
TPA/TPE/FP/FN		114/24/163/69	120/6/15/36	126/6/12/33	144/1/4/23	142/4/9/17	51/7/49/45	94/16/101/68
F1 score	Bottom-level ingredients	73.8 [69.9, 77.2]	87.3 [83.0, 90.4]	90.9 [86.8, 93.4]	91.1 [87.2, 93.8]	93.0 [89.8, 95.1]	48.1 [43.1, 53.0]	77.5 [74.2, 80.3]
Precision		73.5 [69.4, 76.9]	87.3 [82.9, 90.3]	90.8 [86.6, 93.3]	91.1 [87.2, 93.8]	93.0 [89.7, 95.1]	62.9 [58.0, 67.2]	77.2 [73.7, 80.0]
Recall		74.2 [70.3, 77.6]	87.4 [83.1, 90.4]	91.0 [86.9, 93.6]	91.2 [87.2, 93.8]	93.0 [89.8, 95.1]	38.9 [33.5, 44.4]	77.8 [74.6, 80.6]
TPA/TPE/FP/FN		2444/839/44/10	2878/404/15/11	2997/287/18/9	3002/278/16/13	3064/215/14/14	1281/731/26/1281	2563/726/33/4
F1 score	Nesting	66.8 [61.7, 71.6]	88.1 [82.4, 91.4]	90.8 [87.3, 93.6]	92.2 [88.6, 94.6]	94.1 [90.8, 96.2]	62.6 [55.3, 69.0]	72.5 [67.1, 77.0]
Precision		56.0 [49.5, 62.1]	87.6 [76.8, 92.8]	90.5 [85.9, 94.0]	90.4 [84.8, 94.0]	93.8 [89.0, 96.7]	59.3 [49.7, 67.3]	67.5 [60.1, 73.2]
Recall		83.0 [76.9, 87.7]	88.5 [83.4, 92.3]	91.1 [86.4, 94.4]	94.0 [90.3, 96.4]	94.4 [90.9, 96.8]	66.3 [57.3, 74.8]	78.4 [72.0, 83.5]
TPA/FP/FN		258/203/53	269/38/35	277/29/27	283/30/18	287/19/17	108/74/55	243/117/67

True positive accurate (TPA), true positive error (TPE), false positive (FP), and false negative (FN)

Supplementary Table 7: Ingredient mapping performance of GPT-4.1 mini with varying training data sizes (0/5 = no fine-tuning, 4/5 = 5-fold LLM) vs. non-fine-tuned GPT-4.1 nano and GPT-4.1

Metric	Sub-area	GPT-4.1 mini					GPT-4.1 nano	GPT-4.1
		**0/5 No fine-tuning**	**1/5 fine-tuning set size**	**2/5 fine-tuning set size**	**3/5 fine-tuning set size**	**4/5 fine-tuning set size**	**No fine-tuning**	**No fine-tuning**
Exact match (%)	Ingredient name	74.6 [71.0, 77.8]	79.9 [76.5, 83.0]	82.0 [78.9, 84.9]	86.5 [83.6, 89.1]	84.4 [81.4, 87.0]	64.3 [60.5, 68.1]	78.9 [75.5, 82.0]
Levenshtein		3.1 [2.5, 4.2]	2.4 [1.9, 3.5]	2.0 [1.5, 3.1]	1.5 [1.1, 2.8]	1.7 [1.3, 2.9]	6.4 [5.1, 8.3]	2.8 [2.2, 3.9]
Vector distance		4.5 [3.9, 5.4]	3.0 [2.5, 3.6]	2.5 [2.1, 3.1]	1.9 [1.5, 2.4]	2.2 [1.8, 2.8]	7.0 [6.1, 8.0]	3.8 [3.1, 4.5]
F1 score	Ingredient name (retrieval only)	79.4 [76.1, 82.3]	85.1 [82.2, 87.6]	87.5 [84.8, 89.8]	90.5 [88.2, 92.4]	90.2 [87.7, 92.1]	69.1 [65.3, 72.6]	83.8 [80.9, 86.4]
Precision		75.7 [72.2, 79.0]	83.2 [79.9, 86.1]	85.4 [82.4, 88.2]	89.4 [86.6, 91.7]	89.3 [86.5, 91.7]	66.3 [62.3, 69.9]	79.6 [76.3, 82.7]
Recall		83.5 [80.2, 86.4]	87.1 [84.1, 89.7]	89.6 [86.8, 92.0]	91.6 [89.0, 93.7]	91.1 [88.4, 93.3]	72.2 [68.3, 75.9]	88.5 [85.8, 91.0]
TPA/TPE/FP/FN		459/82/65/9	479/47/50/24	493/40/44/17	504/26/34/20	501/27/33/22	397/138/64/15	487/60/65/3

True positive accurate (TPA), true positive error (TPE), false positive (FP), and false negative (FN)

Supplementary Table 8: Parsing-to-mapping performance of GPT-4.1 mini with varying training data sizes (0/5 = no fine-tuning, 4/5 = 5-fold LLM) vs. non-fine-tuned GPT-4.1 nano and GPT-4.1

**Metric**	**Sub-area**	GPT-4.1 mini					GPT-4.1 nano	GPT-4.1
		**0/5 No fine-tuning**	**1/5 fine-tuning set size**	**2/5 fine-tuning set size**	**3/5 fine-tuning set size**	**4/5 fine-tuning set size**	**No fine-tuning**	**No fine-tuning**
Exact match (%)	Ingredient list	20.2 [16.1, 24.8]	41.3 [36.0, 46.9]	43.8 [38.2, 49.4]	47.5 [42.0, 53.1]	50.0 [44.7, 55.6]	20.2 [16.1, 24.8]	27.6 [23.0, 32.6]
DeepDiff		4.8 [4.2, 5.5]	2.0 [1.7, 2.4]	1.7 [1.4, 2.0]	1.4 [1.1, 1.8]	1.3 [1.1, 1.6]	5.9 [5.2, 6.7]	3.9 [3.3, 4.5]
Exact match (%)	Ingredient names	78.1 [74.3, 81.1]	86.2 [82.1, 89.0]	88.2 [84.2, 90.4]	88.6 [84.9, 91.0]	90.1 [87.1, 92.0]	73.3 [68.4, 76.8]	83.2 [79.9, 85.8]
Levenshtein		3.1 [2.6, 3.8]	2.1 [1.5, 3.3]	1.6 [1.1, 2.9]	1.5 [1.1, 2.2]	1.3 [0.9, 1.8]	4.4 [3.6, 5.5]	2.5 [2.1, 3.2]
Vector distance		5.6 [4.6, 7.1]	3.4 [2.4, 5.0]	2.8 [2.0, 4.4]	2.9 [2.0, 4.3]	2.4 [1.7, 3.5]	7.0 [5.7, 8.9]	4.1 [3.3, 5.3]
F1 score	Bottom-level ingredients	77.5 [73.6, 80.6]	85.8 [81.7, 88.7]	87.8 [83.8, 90.2]	88.2 [84.4, 90.8]	89.7 [86.6, 91.7]	55.3 [50.2, 60.2]	82.7 [79.4, 85.4]
Precision		77.1 [73.1, 80.2]	85.8 [81.6, 88.7]	87.7 [83.7, 90.1]	88.2 [84.4, 90.7]	89.7 [86.5, 91.7]	72.3 [67.4, 76.0]	82.3 [78.9, 85.1]
Recall		77.9 [74.0, 80.9]	85.9 [81.8, 88.7]	87.9 [83.9, 90.3]	88.2 [84.5, 90.8]	89.7 [86.7, 91.7]	44.8 [38.9, 50.6]	83.1 [79.8, 85.7]
TPA/TPE/FP/FN		2565/718/44/10	2828/454/15/11	2895/389/18/9	2906/374/16/13	2954/325/14/14	1474/538/26/1281	2735/554/33/4

True positive accurate (TPA), true positive error (TPE), false positive (FP), and false negative (FN)
